# Eye Disorders in the Post-COVID Era

**DOI:** 10.18502/jovr.v16i4.9740

**Published:** 2021-10-25

**Authors:** Seyed-Hossein Abtahi, Hosein Nouri, Siamak Moradian, Shahin Yazdani, Hamid Ahmadieh

**Affiliations:** ^1^Ophthalmic Research Center, Research Institute for Ophthalmology and Vision Science, Shahid Beheshti University of Medical Sciences, Tehran, Iran; ^2^Ophthalmic Epidemiology Research Center, Research Institute for Ophthalmology and Vision Science, Shahid Beheshti University of Medical Sciences, Tehran, Iran

The COVID-19 pandemic has substantially affected all aspects of healthcare, and these effects are still unfolding. COVID-19 has caused ocular problems both directly and indirectly; further causes of concern are side effects of COVID-19 treatment and indirect consequences of lockdowns and shelter-in-place orders. Despite the ongoing evolution of the coronavirus due to constant mutations,^[1]^ the pandemic will eventually come to a relative end or at least slowdown, and we must prepare for what lies ahead. In this editorial, we will discuss the probable impacts of the pandemic on ophthalmic disorders and surgical indications over the coming years.

Various ocular manifestations have been reported in COVID-19 cases, which involve different structures, including the eyelids, conjunctiva, cornea, episclera, retina, and cranial nerves. Most retinal pathologies associated with COVID-19 have roots in vascular complications such as retinal vasculitis and retinal arteries and veins occlusions.^[2]^ Other causes of concern are potential adverse effects of COVID-19 medications such as anticoagulants.^[3]^ Furthermore, patients' routine use of face masks may theoretically increase the risk of infections, as the exhalate is directed toward the periocular area during procedures such as intravitreal injections.^[4,5]^

COVID-19 has also indirectly affected ophthalmology patients. The ophthalmology practice necessitates close, in-person visits and carries a high risk of viral transmission.^[6]^ Therefore, follow-up protocols and visit routines have been modified worldwide, particularly earlier in the pandemic, to reduce the risk of disease transmission.^[7]^ The postponement of in-person follow-up visits and replacement with tele/virtual visits may predispose to delayed or wrong diagnoses and lead to insufficient treatment.^[8]^

Most hospitalized COVID-19 patients receive systemic corticosteroids to alleviate systemic inflammation and prevent pulmonary fibrosis; dexamethasone is widely administered and has been shown to reduce mortality in patients receiving oxygen support or mechanical ventilation.^[9]^ Whatever their efficacy in the setting of COVID-19, steroids have established causal links with several long-term ocular complications, including cataracts and glaucoma.^[10]^ The associated risk tends to rise in a dose- and duration-dependent fashion.^[11]^ Dexamethasone, in particular, is more potent than prednisolone and has a greater propensity to cause glaucoma and cataracts.^[12]^ It is well-known that dexamethasone induces nuclear and posterior subcapsular cataracts.^[13,14]^ With the widespread use of steroids, we will probably witness a rise in cataract rates, especially early-onset cases, in the forthcoming decade leading to a decrease in the mean age of patients requiring cataract surgery. It is well documented that younger patients undergoing phacoemulsification are more prone to vitreoretinal problems;^[15]^ the result may be an increase in the number of vitreoretinal surgeries.

Glucocorticoids can affect the trabecular meshwork and reduce outflow facility leading to increased intraocular pressure.^[16]^ Genetic predisposition plays a pivotal role in this regard; while some individuals are more susceptible to steroid-induced complications, that is, steroid responders who constitute an estimated 37–39% of the general population, some are among steroid non- responders.^[17]^ Previously diagnosed glaucoma patients are also at risk of exacerbation following steroids administration.^[10]^ Hence, the utilization of systemic steroids due to COVID-19 may increase the number of glaucoma cases in need of treatment which, in turn, translates to a higher demand for antiglaucoma medications and glaucoma surgery.

Glucocorticoids also adversely affect glycemic control in patients with diabetes mellitus (DM). This correlation is well documented, and decreased insulin sensitivity is the primary mechanism.^[18]^ High-dose dexamethasone has been documented to reduce insulin secretory capacity as well.^[19]^ New-onset DM is also frequently observed with steroid use; the risk is more pronounced with higher doses and longer durations of treatment.^[20]^ With the anticipated growth in the number of new DM cases and progression in established ones, a significant increase in the incidence of DM complications, such as cataracts and diabetic retinopathy may be expected in the years to come. This will ultimately increase the need for cataract surgery on the one hand and lasers, intravitreal injections, and vitreoretinal surgery for diabetic retinopathy on the other hand.

Diagnostic radiologic modalities emitting ionizing radiation have been extensively employed during the COVID-19 pandemic; many hospitalized patients may undergo multiple imaging sessions during their stay.^[21]^ The carcinogenic potential of cumulative low dose radiation (i.e., < 100 mGy) remains controversial. However, a recent meta-analysis calculated an overall excessive relative risk of 0.03 (95% CI = 0.07 to 0.25) and 0.16 (95% CI = 0.37 to 5.32) for solid tumors and leukemia, respectively, associated with < 100 mGy cumulative irradiation doses in adults.^[22]^ Of note, the thyroid is exposed to radiation during chest imaging sessions, which is a presumed risk factor for the development of Graves' ophthalmopathy.^[23]^ With insufficient data on imaging protocols and cumulative exposure to ionizing radiation, any prediction regarding the incidence of malignancies with ophthalmic manifestations in the future remains speculative. Multi-center surveys are warranted to determine if such risks are significant enough to be prospectively investigated among hospitalized COVID-19 survivors.

Prolonged lockdowns, stay-home recommendations, and school closures have expectedly had substantial effects on the duration of interaction with digital screens and video games among school-age children.^[24]^ Some cultural and educational framework changes may probably be favored and adhered to even after the pandemic. Altogether, with increased time spent on digital screens and near-work tasks, and restricted outdoor activities, especially among the pediatric population, an increased incidence of myopia and myopic progression may be encountered in the future.^[25]^ In one or two decades, we may witness an increased demand for refractive surgery^[26]^ and a greater need for vitreoretinal treatment for myopia complications.

The majority of the issues discussed above point toward an exponential increase in the demand for ophthalmic surgery, especially vitreoretinal treatments. The additional expenses involved will impose a burden on healthcare systems and insurance companies.^[27]^ All nations dealing with large numbers of COVID cases, especially those with developing economies, will suffer.

In summary, the huge morbidity and death toll imposed by the pandemic has led to the adoption of life-saving treatment protocols with the downside of complications. Our concerns highlight the need for modification of unnecessary or overuse of corticosteroids. Physicians should consider the inherent risks of high-dose corticosteroid use on the whole human body, including ocular side effects such as cataract formation. In individuals with a personal or familial background of DM or glaucoma, physicians should weigh the pros and cons of corticosteroid administration. The community should also be aware of the increased risk of childhood myopia due to stay-at-home orders and excessive near-vision tasks.

##  Financial Support and Sponsorship 

No funding was received for the preparation of this article.

##  Conflicts of Interest

The authors declare no financial or proprietary interests in any material discussed in this article.
